# The Effectiveness of a Smartphone Application on Modifying the Intakes of Macro and Micronutrients in Primary Care: A Randomized Controlled Trial. The EVIDENT II Study

**DOI:** 10.3390/nu10101473

**Published:** 2018-10-10

**Authors:** Jose I. Recio-Rodriguez, Cristina Agudo Conde, Maria J. Calvo-Aponte, Natividad Gonzalez-Viejo, Carmen Fernandez-Alonso, Nere Mendizabal-Gallastegui, Beatriz Rodriguez-Martin, Jose A. Maderuelo-Fernandez, Emiliano Rodriguez-Sanchez, Manuel A. Gomez-Marcos, Luis Garcia-Ortiz, On Behalf of the EVIDENT Investigators

**Affiliations:** 1 Primary Health Care Research Unit, The Alamedilla Health Center, Castilla and León Health Service (SACYL), Institute of Biomedical Research of Salamanca (IBSAL), Spanish Research Network for Preventive Activities and Health Promotion in Primary Care (REDIAPP), 37003 Salamanca, Spain; cagudoconde@yahoo.es (C.A.C.); jmaderuelo@saludcastillayleon.es (J.A.M.-F.); emiliano@usal.es (E.R.-S.); magomez@usal.es (M.A.G.-M.); Lgarciao@usal.es (L.G.-O.); 2 Faculty of Health Sciences, Universidad de Burgos, 09292 Burgos, Spain; 3 Primary Health Care Research Unit of Barcelona, Primary Healthcare University Research Institute IDIAP-Jordi Gol, 08007 Barcelona, Spain; mjoseaponte@hotmail.com; 4 Torre Ramona Health Center, Aragón Health Service, 50013 Zaragoza, Spain; natigonviejo@gmail.com; 5 Casa de Barco Health Center, Castilla y León Health Service, 47007 Valladolid, Spain; carmenferal@gmail.com; 6 Primary Care Research Unit of Bizkaia, Basque Health Service-Osakidetza, 48014 Bilbao, Spain; NERE.MENDIZABALGALLASTEGUI@osakidetza.net; 7 Río Tajo Health Center, Castilla-La Mancha Health Service. University of Castilla-La Mancha, 13071 Talavera de la Reina, Spain; Beatriz.RMartin@uclm.es; 8 Department of Medicine, University of Salamanca, 37008 Salamanca, Spain; 9 Biomedical and Diagnostic Sciences Department, University of Salamanca, 37008 Salamanca, Spain; 10 Spanish Research Network for Preventive Activities and Health Promotion in Primary Care, 08025 Barcelona, Spain; Laalamedilla@gmail.com

**Keywords:** diet, smartphone applications, diet records, dietary fats, dietary carbohydrates, general population

## Abstract

Background: This study evaluates the effectiveness of adding a diet smartphone application to standard counseling to modify dietary composition over the long term (12 months). Methods: A randomized, controlled, multicenter clinical trial was conducted involving the participation of 833 subjects from primary care clinics (415 to the intervention (counseling + application) group (IG) and 418 to the control (counseling) group (CG)). Both groups were counseled about a healthy diet and physical activity. For the 3-month intervention period, the IG was also trained to use a diet smartphone application that involved dietary self-monitoring and tailored feedback. Nutritional composition was estimated using a self-reported food frequency questionnaire. Results: An analysis of repeated measures revealed an interaction between the group and the percentages of carbohydrates (*p* = 0.031), fats (*p* = 0.015) and saturated fats (*p* = 0.035) consumed. Both groups decreased their energy intake (Kcal) at 12 months (IG: −114 (95% CI: −191 to −36); CG: −108 (95% CI: −184 to −31)). The IG reported a higher percentage intake of carbohydrates (1.1%; 95% CI: 0.1 to 2.0), and lower percentage intakes of fats (−1.0%; 95% CI: −1.9 to −0.1) and saturated fats (−0.4%; 95%CI: −0.8 to −0.1) when compared to the CG. Conclusions: Better results were achieved in terms of modifying usual diet composition from counseling and the diet smartphone application compared to counseling alone. This was evaluated by a self-reported questionnaire, which indicated an increased percentage intake of carbohydrates, and decreased percentage intakes of fats and saturated fats.

## 1. Introduction

Modifications to the composition of macro and micronutrient intakes in the diet have been explored to determine their effects on cardiovascular disease risk [1,2]. More recently, research has considered this in regard to other health outcomes, such as the quality and quantity of sleep [3,4], cognitive decline, and cognitive functions [5]. The most studied dietary pattern has been the Mediterranean diet, which has shown beneficial effects, such as a lower incidence of major cardiovascular events among persons at high risk for cardiovascular conditions [6] and a lower risk for certain types of cancer [7,8]. Changes in macronutrient intake indicate a progressive trend towards a decrease in carbohydrate intake at the expense of an increase in the consumption of total fat and other unhealthy fat subtypes as well as an increased protein intake [9,10,11].

Interventions related to the consumption of macronutrients for health have focused on the restriction of total and saturated fat intakes with the objective of reducing the concentrations of LDL cholesterol and reducing the cardiovascular risk. Recognition of the importance of different types of fat (saturated, polyunsaturated, monounsaturated, or trans fatty acids) and the importance of replacing the consumption of saturated fats with increases in unsaturated fats or carbohydrates have appeared as new targets in dietary management [1].

There has been increasing use and acceptability of smartphone applications to address healthy lifestyles and to support healthy food choices [12,13,14]. However, the documented health outcomes are limited, although some studies have shown a positive effect on the cardiometabolic profile [15] and the diet quality score [16]. The use of smartphone applications has been associated with greater weight loss [17,18,19] but has not shown significant effects on nutrition goals [20,21]. There is little evidence on the potential effects of using new technologies such as smartphone applications to modify dietary patterns and dietary composition. The review by Shoeppe et al. [22] concluded that there is modest evidence that interventions based on mobile technology can improve the diet. Of the studies included in this review, none analyzed the effect on macro and micronutrient intakes. Although some obtained results favorable to the intervention group for the consumption of vegetables [23,24,25] and fruits [26], other research did not find significant effects [21,27,28,29,30]. In general, these studies included a small number of participants with a young average age.

A recent review on the characteristics of smartphone applications for nutrition improvement in the community setting suggests that developed applications should be oriented towards specific target users based on age or sociodemographic characteristics, although this may affect the proliferation of specific applications [31]. Achieving this will require applications to incorporate a detailed registration of foods, goals, and customizable challenges in their design [31]. The EVIDENT II study included a smartphone application involving dietary self-monitoring and tailored feedback which provided necessary evidence to recommend these applications to the general population.

The objective of this study is to evaluate the long-term (12 months) effectiveness of adding a diet smartphone application to standard counseling to modify dietary composition (macro and micronutrients and food groups).

## 2. Materials and Methods

### 2.1. Design Overview

We conducted a national, multicenter, randomized, controlled clinical trial with two parallel groups in six Spanish primary care centers with a 12-month follow-up assessment as part of the EVIDENT II study (ClinicalTrials.gov Identifier: NCT02016014) [32]. 

### 2.2. Procedures

**Figure 1:**
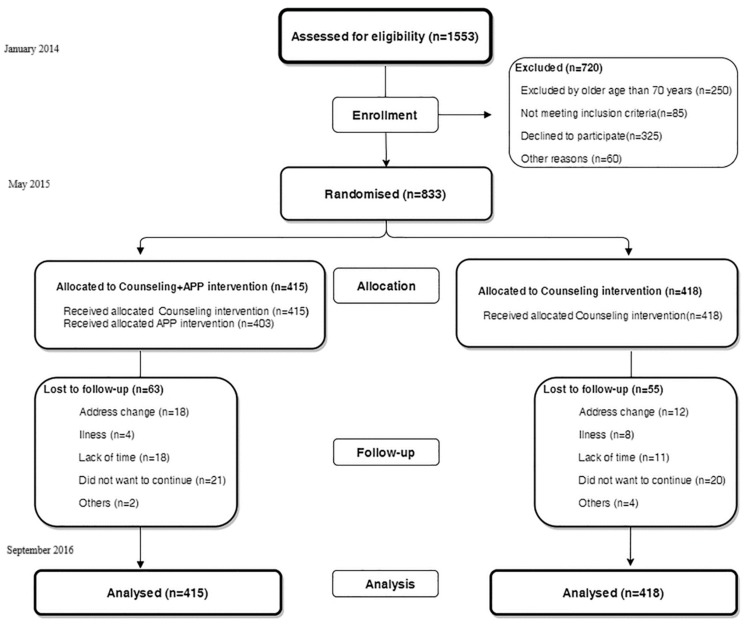
Study flow diagram: enrollment of the participants and completion of the study.

Each participant completed an initial visit and two follow-up visits at three and twelve months after study initiation between January 2014 and September 2016 (Figure 1). 

### 2.3. Setting and Participants

The study included six primary care groups in the Spanish Research Network for Preventive Activities and Health Promotion in Primary Care (REDIAPP). The study population was selected from the EVIDENT I study, whose objective was to analyze the relationship of physical activity and dietary pattern to the blood pressure circadian pattern, pulse wave velocity, and carotid intima-media thickness in individuals without arteriosclerotic disease [33]. The participants were selected by random sampling among the patients who attended a consultation with their family doctor in each participating center. Eligibility criteria included being aged between 18 and 70 years old. Subjects were excluded if they were unable to do exercise or follow the Mediterranean diet, or if they met any of the exclusion criteria of the EVIDENT I study. These criteria were the known presence of coronary or cerebrovascular atherosclerotic disease; heart failure; moderate or severe chronic obstructive pulmonary disease; musculoskeletal disease involving limited walking; advanced respiratory, renal, or hepatic disease; severe mental disease; or a treated oncological disease diagnosed in the last 5 years.

### 2.4. Screening and Randomization

From the 1553 subjects recruited in the EVIDENT I study, 250 were excluded for being older than 70 years, 85 did not meet the inclusion criteria, 325 declined to participate, and 60 were excluded for other reasons. The final group included 833 subjects (Figure 1). The recruited subjects were randomized into two groups with a ratio of 1:1 on the basis of centralization from Salamanca. This included a counseling + application group (intervention group; IG) comprising 415 subjects and a counseling group (control group; CG) with 418 subjects. The allocation sequence was generated through a computer program (Consellería de Sanidade, X.d.G., Epidat 4.0, Santiago de Compostela, Spain) [34] by an independent researcher and was concealed until the trial arms had been assigned.

To minimize contamination between groups, the investigator who performed the intervention was different from the investigator who conducted the evaluation. The investigator who performed the data analysis was blinded. Due to the nature of the study, the subjects could not be blinded to the intervention.

### 2.5. Intervention 

#### 2.5.1 Standardized Nutritional Counseling

A research nurse performed one-to-one standardized counseling geared towards adopting a healthy diet pattern (Mediterranean diet) and complying with the international recommendations on physical activity to both groups. This advice lasted approximately 30 min, and at the end of the session, printed support material (a leaflet) was delivered with a summary of the content covered. The first part of the counseling was aimed at developing the concept of the Mediterranean diet; the second part was aimed at developing the characteristics of good adherence to the Mediterranean diet; and the last part was dedicated to solving and clarifying possible doubts. Participants were given advice taking into account the motivation stage for the change they were in, following the model of Prochaska and Diclemente [35], reinforcing their attitude in case of preparation (subjects that seriously consider the intention to change their behavior in the near future (within 30 days)), action (subjects that are actively working in behavioral changes that affect their health) or maintenance (subjects adopt the behaviors acquired as routine) and encouraging those who were in pre-contemplation (subjects are aware that certain behaviors are a risk for their health or that they have a health problem) and contemplation (subjects are aware that certain behaviors are a risk for their health or that they have a health problem, and agree to make changes within 6 months).

#### 2.5.2 The Counseling + Application Group (IG) Intervention

**Figure 2:**
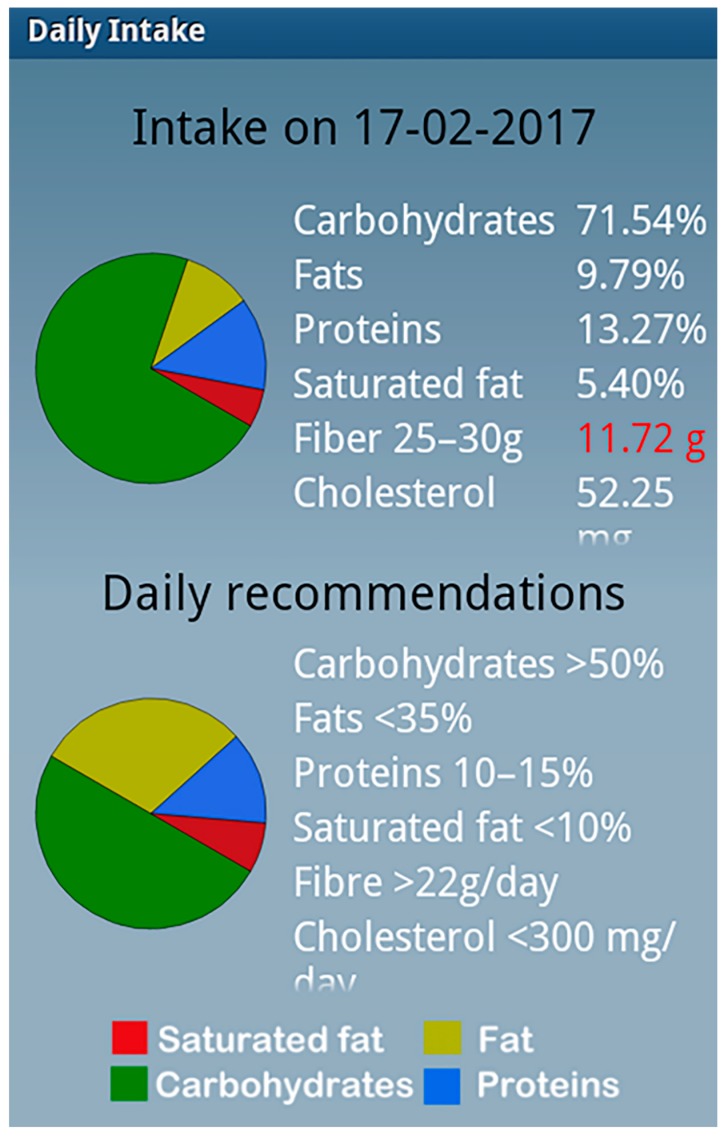
Screenshot of the personal recommendations made by the EVIDENT II diet application.

The IG completed two additional appointments. The first one occurred seven days after the baseline in which the application was explained and the second one was a phone call 15 days after the baseline visit to confirm that the application was being used correctly and to clarify any possible doubts. The application was designed by software engineers in collaboration with dietitians and physical activity experts with an easy-to-use interface. Participants in the IG used a loaned mobile phone which included the diet smartphone application. Another investigator (different from the investigator who performed the standardized counseling) instructed the participants about the use of the EVIDENT II application, which included dietary self-monitoring and tailored feedback through notifications about compliance with dietary recommendations. During the intervention visit, the diet smartphone application was configured with the characteristics of each participant (age, sex, weight and height). The subjects were required to enter their food intake daily (breakfast, lunch, afternoon snack, and dinner) and select dishes and foods from the application menu. The application estimates daily energy and nutrient intake using food composition tables and serving sizes provided by the user. Based on the entered intake characteristics and the provision of adequate proportions of macronutrients, personalized recommendations were produced (Figure 2). Lastly, the final daily summary was reviewed with a balance of food intake, and the device offered a recommended plan for the following days. The application uses an included pedometer to count the individual’s steps every 24 h. The application also analyzes the physical activity reported by the subject during physical activities during which the device cannot be used (for example, swimming and other sports), and evaluates compliance with the objectives of the exercise and provides recommendations to increase activity. The intervention period was 3 months, with long-term effectiveness assessed after 12 months to assess maintenance of these behaviors. The smartphone was returned after three months. The information that remained on the phone was transferred via WiFi to a central computer for subsequent analysis. 

### 2.6. Main Outcomes

The nutritional composition of the usual dietary intake was recorded using a self-administered, semi-quantitative food frequency questionnaire (FFQ). This questionnaire has been validated for a Spanish population [36]. After receiving instructions from the study staff, participants indicated the frequency with which they had consumed each food product in the past year using a 9-item scale (never or almost never, 1–3 times monthly, once weekly, 2–4 times weekly, 5–6 times weekly, once daily, 2–3 times daily, 4–6 times daily, or more than 6 times daily). This questionnaire allows for the estimation of the daily intakes of energy and macro and micronutrients.

### 2.7. Other Measurements

In 2014, the protocol of the EVIDENT II study was published, which describes how the clinical data were collected, the anthropometric measurements were made, and the analytical parameters were obtained [32].

At baseline, we classified the patients according to their motivation for diet change following the model of Prochaska and Diclemente [35].

### 2.8. Ethics Approval and Consent to Participate

The study was approved by the clinical research ethics committee (CEIC) of the health care area of Salamanca (“CEIC of Area de salud de Salamanca”, 21 June 2013) as a coordinating center. It was also approved by the ethics committees of the five collaborating centers (“CEIC of Aragón (CEICA), CEIC of IDIAP Jordi Gol, CEIC of Euskadi (CEIC-E), CEIC of the Castilla la Mancha, and CEIC of the Area de salud de Valladolid Oeste”). Subjects signed informed consent forms prior to inclusion in the study in accordance with the Declaration of Helsinki.

### 2.9. Statistical Analysis

The sample size was estimated for the main study endpoints. The recruitment of 833 subjects, with 415 subjects in the first group and 418 in the second, was considered sufficient to recognize a statistically significant difference of 1.5 percentage points in the total dietary fat intake between groups with an alpha risk level of 0.05 in a two-sided test, a common standard deviation of 6.5%, and a statistical power of 90%. The results are expressed as the mean ± standard deviation for quantitative variables or using the frequency distribution for qualitative variables. The results were analyzed on an intent-to-treat basis. The intention-to-treat analyses was performed with the baseline value carried forward and included all randomized patients in the group to which they were randomly assigned, regardless of their adherence to the application, withdrawal or deviation from the protocol, and anything that happened after randomization [37]. No data imputation was made for missing FFQ data. The student *t*-test was used to compare the means between the two groups, and the paired *t*-test was used to assess changes within the same group.

To analyze the effects of the intervention, we compared the changes observed at 3 and 12 months between the IG and CG by ANCOVA with adjustments for baseline measures of each variable. To compare the data between groups over time, an analysis of variance was performed with repeated measures. The presence or absence of sphericity was taken into account, and the Greenhouse–Geisser correction was carried out. Contrasting hypotheses were established using α = 0.05. The data were analyzed using IBM SPSS Statistics for Windows, Version 23.0 (IBM Corp, Armonk, NY, USA). A value of *p* < 0.05 was considered statistically significant. 

## 3. Results

### 3.1. Baseline Characteristics of Participants and Follow-up

The participants were 60% women (*n* = 249) in the IG and 64% women (*n* = 268) in the CG, with mean ages of 51.4 (12.1) and 52.3 (12.0) years, respectively (*p* > 0.05). No differences were found in the other demographic and clinical characteristics (work situation, educational level, smoking, or physical activity; Table 1). Of the 833 subjects included in the study, 63 (15.1%) in the IG and 55 (13.1%) in the CG were not included in the follow-up for the reasons detailed in Figure 1. A total of 715 subjects, 352 in the IG and 363 in the CG, completed the 12-month assessment. No differences were found between the groups in the dietary habits stage of change analysis. The group of participants who did not complete the study (12-month visit) were younger, included a higher proportion of students and unemployed individuals and declared less physical activity than those who completed the entire study (Supplementary Material: Table S1).

### 3.2. Changes in the Intakes of Macro and Micronutrients

**Figure 3:**
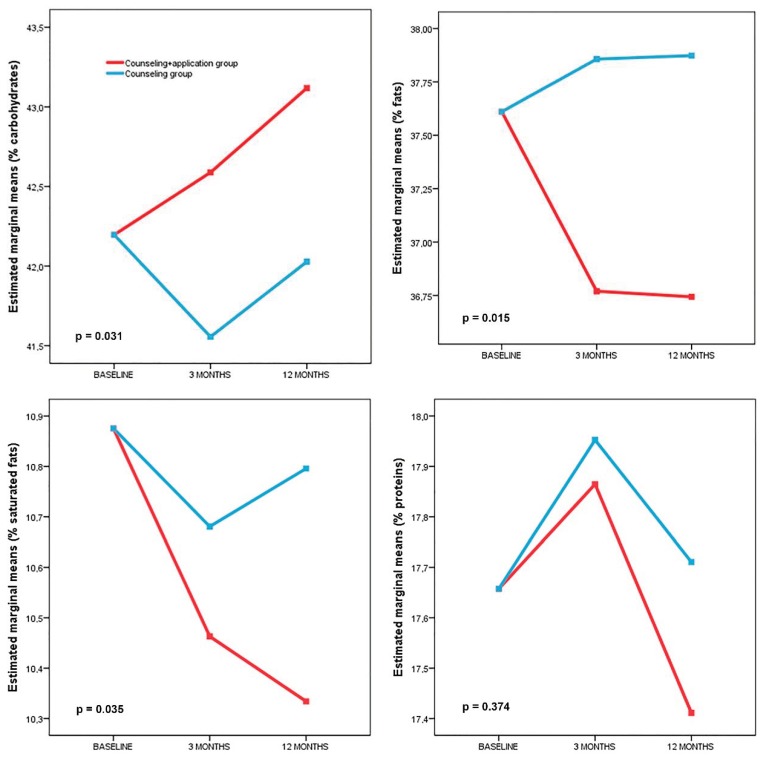
Changes in macronutrients after randomization by group (repeated measures analysis).

The changes in macronutrient intake after randomization are presented in Figure 3. Repeated measures ANOVA revealed a significant interaction between the group (IG or CG) and the percentage intakes of carbohydrates (*p* = 0.031), total dietary fat (*p* = 0.015) and saturated fat (*p* = 0.035) of the total intake. No interactions were found between the other variables, including energy intake (*p* = 0.148), or percentage intakes of protein (*p* = 0.374), polyunsaturated fatty acids (*p* = 0.450), or fiber (*p* = 0.163).

Self-reported information about the caloric and nutritional content of the diet indicated a similar decrease in energy intake (Kcal) at 12 months in both study groups (IG: −114 (95% CI: −191 to −36); CG: −108 (95% CI: −184 to −31)). Regarding the macronutrient composition of the usual diet, the IG showed decreased percentage intakes of total fat (−1.0% (95% CI: −1.8 to −0.1)) and saturated fat (−0.6% (95% CI: −0.9 to −0.3)) at 12 months with respect to the initial percentage intakes, with an increase in the percentage intake of carbohydrates (1.0% (95% CI: 0.1 to 1.9)). The CG showed a decrease in the percentage intake of carbohydrates (−0.8% (95% CI: −1.5 to −0.1)) at 3 months, but none of these parameters were modified at the 12-month follow up assessment.

In relation to the micronutrient composition, both groups showed decreased trans-fat (g/day) and cholesterol intakes (mg/day) at 3 and 12 months, but only the IG showed increased consumption of folate (15.1 µg/day (95% CI: 0.2 to 30.0)) and β-carotene (388 µg/day (95% CI: 79 to 697)). However, this difference disappeared at 12 months. In the IG, there was a decrease in the intake of ω-6 (g/day) and calcium (mg/day) at 12 months, as shown in Table 2.

At 12 months, a beneficial effect was observed in the IG with respect to the control group for the percentage intake of carbohydrates(1.1% (95% CI: 0.1 to 2.0)) with lower percentage intakes of total fat (−1.0% (95% CI: −1.9 to −0.1)) and saturated fat (−0.4% (95% CI: −0.8 to −0.1)), and a lower intake of trans-fats (−0.07 g/day (95% CI: −0.12 to −0.01)), as shown in Table 3. 

### 3.3. Changes in the Intake of Food Groups

At the 12-month follow up assessment, both groups had decreased their intakes of red meat (IG: −8.2 g/day (95% CI: −13.5 to −3.0); CG: −5.7 g/day (95% CI: −10.4 to −1.0)) and processed meat (IG: −9.1 g/day (95% CI: −11.9 to −6.2); CG: −3.0 g/day (95% CI: −5.7 to −0.4)). However, only the CG had significantly decreased their intake of ready-made food (−2.0 g/day (95% CI: −3.9 to −0.2)), as shown in Table 4. 

The results show a beneficial effect of the intervention through a greater decrease in the consumption of processed meat (g/day) in the IG (−4.3 (95% CI: −7.1 to −1.5)) than in the CG. The rest of the food groups did not show differences between groups (Table 5).

## 4. Discussion 

There have been few randomized and controlled clinical trials that have analyzed the effects of using new technologies to modify the nutritional composition of the usual diet. The EVIDENT II study provides relevant information in this regard with a large sample of healthy adults. The present intervention combined standardized counseling with the use of a diet smartphone application that incorporates personalized recommendations, and this combination resulted in effective changes in the composition of the usual diet, as measured by self-reported information. These results included an increase in the percentage intake of carbohydrates (coming from complex carbohydrates, pulses, nuts, and vegetables) and a decrease in the percentage intakes of total fat and saturated fat, in addition to a reduction in the intake of-trans fats and the consumption of processed meats.

Few studies have looked at the impacts of variation in macronutrient consumption on cardiovascular health. However, previous studies agree that variations in the proportions of fat and carbohydrate intake in the diet within the relatively narrow ranges recommended by different nutritional guidelines significantly impact the metabolic and lipid profile (HDL and triglyceride concentrations) and markers of low-grade inflammation [38,39]. The consumption of saturated fatty acids has also been inversely associated with the mortality rate due to cerebrovascular disease [40]. In the EVIDENT II study, the difference between the two groups in terms of macronutrient intake was relatively small, although there were statistically and clinically significant differences. However, beneficial effects of the intervention on other clinical variables have been found, for example, reductions in abdominal obesity and the percentage of body fat, especially in women [41].

This evidence supports the need to design interventions such as the one analyzed in the EVIDENT study (counseling plus the use of a diet smartphone application), which have been shown to increase the percentage intake of carbohydrates (1.1%) and decrease the percentage intakes of total fats (1.0%) and saturated fats (0.4%) in the diet. In a recent review on this topic, Coughlin et al. [17] concluded that the use of smartphone applications in randomized trials is associated with better dietary compliance with recommendations to consume lower-calorie, low-fat, and high-fiber foods, which results in more weight loss. However, very few of the studies included in the review obtained significant results in terms of nutrition goals. In one of them, non-significant 6-month reductions of 415 and 468 calories were achieved in single-counseling and counseling-plus-app groups, respectively, and a 4.9% reduction in total fat consumption occurred only in the second group. The findings of the EVIDENT II study found a significant decrease of 1% in total fat consumption, but in a different context from the study performed by Allen et al. [18]. While the EVIDENT II analyzed a large sample of the general population, Allen et al. [18] analyzed data from a small sample of individuals with obesity (*n* = 68). In addition, the EVIDENT studio achieved these results within the specific context of advice on the Mediterranean diet, where the consumption of fat is more permissive when coming from olive oil. In addition, these decreases in fat and saturated fat consumption were accompanied by an increase in the consumption of complex carbohydrates, resulting in a more favorable intake profile of macronutrients. This could have resulted from the personalized recommendations that the EVIDENT II application offers, which are oriented toward the achievement of recommended percentages of macronutrients (Figure 2).

The EVIDENT II study did not obtain favorable results for the intervention group in relation to adherence to the Mediterranean diet over the short-term (3 months) [42]. Likewise, there were no favorable changes to the intervention group in terms of modification of the consumption of food groups. In contrast, in a study by Allman-Farinelli et al. [25] in 250 individuals (aged 18–35 years) where the intervention group used four applications (one for behavior) for 12 weeks, positive results were obtained that were maintained, compared with controls, at 9 months, with greater probabilities of meeting recommendations for fruits (OR 3.83, 95% CI 2.10–6.99) and vegetables (OR 2.42, 95% CI 1.32–4.44). Mummah et al. [23] had similar results in a study involving 17 people (aged 18–50 years) who used an application to promote the consumption of vegetables for 12 weeks. Specifically, they found a difference of 7.4 servings in the application group with respect to the control group. These two works were carried out on young people using applications specifically designed for these purposes. 

The successful use of these health applications could be strengthened by tailoring them to meet personal needs [43]. The use of applications influences the consciousness and self-education about nutrition. Some populations, such as people with diabetes or obesity, may benefit from strategies that combine nutritional counseling and new technologies. However, more clinical trials with broad representative samples are required to determine their potential effects. Future research should clarify the possible effects of age, gender, or educational level on the success of using diet applications to determine the groups of people who are most likely to benefit from the support of new technologies for health promotion.

This study has several limitations. First, the nature of the intervention made it impossible to blind the participants, which may have affected the results. Furthermore, the main results of the study were based on the FFQ, which uses self-reported information on the consumption of certain foods in the usual diet. Moreover, the FFQ used here asks participants about their intake over the past year, so the results at 3 months include the changes in the diet of the 3 months post-randomization and also information on the nutritional composition of the diet from the previous 9 months. The recorded loss rate (near 10%) may have biased the study sample composition to some extent because certain populations may have experienced difficulty using the application and consequently, decided to leave the study. Lastly, although we recommended that participants did not use other applications or wearables that register nutrition and physical activity during the study period, we have no total guarantee that none of the participants used such tools. In addition, it is necessary to point out that that the mobile application was not developed according to a behavioral change theory.

## 5. Conclusions

In summary, the intervention, consisting of nutritional counseling and a diet smartphone application, achieved better results than counseling alone in modifying the diet, as indicated by self-reported caloric and nutritional content information. These results include an increase in the percentage intake of carbohydrates and a decrease in the percentage intakes of total fat and saturated fat, in addition to a reduction in the intake of trans-fats and the consumption of processed meats.

## Figures and Tables

**Table 1:** Baseline characteristics of the study population.

Baseline Characteristics		IG (Advice + Application) (415; 49.8%)		CG (Advice) (418; 50.2%)		
		Mean/*N*	SD/(%)	Mean/N	SD/(%)	*p*-Value
Age (years)		51.4	12.1	52.3	12.0	0.287
Females (*n*, %)		249	(60.0)	268	(64.1)	0.226
Work situation (*n*, %)	Works outside of home	228	(54.9)	203	(48.6)	0.246
	Homemaker	53	(12.8)	72	(17.2)	
	Retired	77	(18.6)	89	(21.3)	
	Student	10	(2.4)	8	(1.9)	
	Unemployed	47	(11.3)	46	(11.0)	
Educational level (*n*, %)	University studies	117	(28.2)	132	(31.6)	0.417
	Middle or high school	208	(50.1)	208	(49.8)	
	Elementary school	90	(21.7)	78	(18.7)	
Smoking (*n*, %)	Non-smoker	190	(45.8)	166	(39.7)	0.203
	Smoker	94	(22.7)	108	(25.8)	
	Former smoker	131	(31.6)	144	(34.4)	
BMI mean (Kg/m^2^)		28.1	5.1	27.6	4.59	0.142
BMI Categories (*n*, %)	BMI < 25	117	(28.2)	131	(31.3)	0.501
	BMI 25–30	172	(41.4)	173	(41.4)	
	BMI > 30	126	(30.4)	114	(27.3)	
Systolic blood pressure (mmHg)		124	16	124	17	0.749
Diastolic blood pressure (mmHg)		76	10	76	10	0.409
Total cholesterol (mg/dL)		202	35	206	37	0.086
Glycated haemoglobin (%)		5,5	0.5	5.5	0.6	0.870
Physical activity						
Counts min/week		69.0	70.4	65.9	69.4	0.539
METS/ min /week		1850.8	891.7	1762.6	922.0	0.177
Dietary habits stage of change, n (%)	Precontemplation	34	8.3	34	8.2	0.910
	Contemplation	26	6.3	20	4.8	
	Preparation	59	14.3	57	13.8	
	Action	18	4.4	18	4.3	
	Maintenance	275	66.7	285	68.8	

IG: intervention group; CG: control group; BMI: body mass index; METS: Metabolic Equivalent of Task.

**Table 2:** Changes in macro and micronutrients from baseline to 3 and 12 months.

Macro/Micronutrients	Baseline		3 Months (Difference)		12 Months (Difference)	
	IG (Advice + Application) Mean ± SD	CG (Advice) Mean ± SD	IG (Advice + Application) Mean (95% CI)	CG (Advice) Mean (95% CI)	IG (Advice + Application) Mean (95% CI)	CG (Advice) Mean (95% CI)
Energy intake (Kcal/day)	2496 ± 753	2450 ± 816	−63 (−137 to 12)	− 138 (−206 to −69) **	−114 (−191 to −36) **	−108 (−184 to − 31) **
Carbohydrate (% of energy)	42.2 ± 6.6	42.5 ± 7.4	0.2 (−0.5 to 1.0)	−0.8 (−1.5 to −0.1) *	1.0 (0.1 to 1.9) *	−0.3 (−1.0 to 0.5)
Protein (% of energy)	17.6 ± 3.2	17.6 ± 3.4	0.2 (−0.2 to 0.6)	0.3 (−0.0 to 0.7)	−0.3 (−0.7 to 0.1)	0.1 (−0.2 to 0.4)
Total fat (% of energy)	37.7 ± 6.4	37.3 ± 6.5	−0.7 (−1.4 to −0.1) *	0.4 (−0.3 to 1.0)	−1.0 (−1.8 to −0.1) *	0.3 (−0.4 to 1.0)
Saturated fat (% of energy)	11.0 ± 2.7	10.8 ± 2.4	−0.4 (−0.7 to −0.1) **	−0.2 (−0.4 to 0.1)	−0.6 (−0.9 to −0.3) **	−0.1 (−0.3 to 0.2)
Monounsaturated fat (% of energy)	16.7 ± 3.6	16.4 ± 3.9	−0.4 (−0.8 to 0.0) *	0.3 (−0.1 to 0.7)	−0.2 (−0.7 to 0.3)	0.4 (−0.1 to 0.8)
Polyunsaturated fat (% of energy)	6.1 ± 2.2	6.2 ± 2.2	0.0 (−0.2 to 0.3)	0.2 (−0.0 to 0.4)	−0.1 (−0.4 to 0.1)	0.0 (−0.2 to 0.3)
Trans fat (g/day)	0.79 ± 0.54	0.74 ± 0.47	−0.10 (−0.14 to −0.06) **	−0.08 (−0.13 to −0.04) **	−0.14 (−0.18 to −0.10) **	−0.06 (−0.11 to −0.01) *
Fiber (g/day)	27.8 ± 10.9	27.9 ± 11.9	0.9 (−0.2 to 1.9)	−0.7 (−1.8 to 0.3)	0.0 (−1.2 to 1.1)	−0.12 (−1.42 to 1.17)
Cholesterol (mg/day)	465.0 ± 183.2	453.1 ± 170.3	−15.8 (−33.7 to 2.0)	−17.6 (−33.4 to −1.9) *	−48.1 (−67.9 to −28.2) **	−24.4 (−42.9 to −6.0) **
Alcohol (g/day)	9.2 ± 13.8	9.0 ± 14.2	0.4 (−0.6 to 1.4)	−0.1 (−1.0 to 0.7)	0.4 (−0.6 to 1.5)	−1.1 (−2.2 to 0.1)
Ω3 (g/day)	1.1 ± 0.6	1.1 ± 0.6	0.0 (−0.1 to 0.1)	0.0 (−0.0 to 0.1)	0.0 (−0.1 to 0.1)	0.0 (−0.1 to 0.1)
Ω6 (g/day)	13.3 ± 7.5	13.2 ± 8.2	−0.5 (−1.3 to 0.3)	−0.4 (−1.2 to 0.4)	−1.1 (−2.0 to −0.2) *	−0.7 (−1.6 to 0.2)
Calcium (mg/day)	1172 ± 409	1144 ± 421	−7 (−47 to 34)	−34 (−71 to 2)	−45 (−89 to −1.0) *	−10 (−54 to 33)
Folate (µg/day)	411.8 ± 146.1	414.8 ± 175.9	15.0 (0 to 30.0) *	−4.8 (−19.8 to 10.1)	0.3 (−15.5 to 16.2)	7.2 (−12.9 to 27.3)
Vitamin C (mg/day)	245.0 ± 124.3	244.9 ± 133.3	5.9 (−6.9 to 18.8)	3.3 (−7.5 to 14.0)	0.9 (−12.6 to 14.3)	3.2 (−11.0 to 17.3)
β-Carotene (µg/day)	3338 ± 2272	3564 ± 2725	387 (78 to 696) *	118 (−161 to 396)	137 (−134 to 407)	94 (−185 to 373)

IG: intervention group; CG: control group; CI: confidence interval; Ω-3: omega 3 fatty acids; Ω-6: omega 6 fatty acids, * *p* < 0.05; ** *p* < 0.01.

**Table 3:** Effect of the intervention in terms of variation in macro and micronutrients.

Macro/Micronutrients	Mean Difference (IG – CG) 3 Months		Mean Difference (IG – CG) 12 Months	
	Mean Difference (CI 95%)	*p*-Value	Mean Difference (CI 95%)	*p*-Value
Energy intake (Kcal/day)	90 (2 to 177)	0.044	1 (−92 to 94)	0.983
Carbohydrate (% of energy)	0.8 (−0.1 to 1.6)	0.091	1.1 (0.1 to 2.0)	0.023
Protein (% of energy)	−0.1 (−0.5 to 0.3)	0.668	−0.4 (−0.8 to 0.1)	0.097
Total fat (% of energy)	−0.9 (−1.7 to 0.0)	0.043	−1.0 (−1.9 to −0.1)	0.022
Saturated fat (% of energy)	−0.1 (−0.5 to 0.2)	0.427	−0.4 (−0.8 to −0.1)	0.007
Monounsaturated fat (% of energy)	−0.5 (−1.0 to 0.0)	0.042	−0.3 (−0.9 to 0.3)	0.292
Polyunsaturated fat (% of energy)	−0.18 (−0.4 to 0.1)	0.222	−0.2 (−0.5 to 0.1)	0.204
Trans fat (g/day)	0.00 (−0.05 to 0.06)	0.812	−0.07 (−0.12 to −0.01)	0.013
Fiber (g/day)	1.58 (0.27 to 2.89)	0.018	0.13 (−1.43 to 1.69)	0.869
Cholesterol (mg/day)	4.3 (−16.2 to 24.7)	0.682	−16.6 (−38.2 to 5.0)	0.131
Alcohol (g/day)	0.6 (−0.6 to 1.8)	0.326	1.3 (−0.1 to 2.7)	0.063
Ω3 (g/day)	−0.0 (−0.1 to 0.1)	0.893	−0.0 (−0.1 to 0.1)	0.436
Ω6 (g/day)	0.0 (−0.9 to 1.0)	0.955	−0.4 (−1.4 to 0.6)	0.402
Calcium (mg/day)	35 (−14 to 83)	0.163	−22 (−76 to 33)	0.435
Folate (µg/day)	20.5 (1.4 to 39.5)	0.035	−5.0 (−28.5 to 18.6)	0.679
Vitamin C (mg/day)	3.8 (−11.4 to 19.0)	0.621	1.1 (−15.8 to 18.0)	0.899
β-Carotene (µg/day)	178 (−202 to 557)	0.358	−3 (−343 to 336)	0.985

IG: intervention group; CG: control group; CI: confidence interval; Ω-3: omega 3 fatty acids; Ω-6: omega 6 fatty acids.

**Table 4:** Changes in food groups from baseline to 3 and 12 months.

Food Group	Baseline		3 Months (Difference)		12 Months (Difference)	
	IG (Advice + Application) Mean ± SD	CG (Advice) Mean ± SD	IG (Advice + Application) Mean (95% CI)	CG (Advice) Mean (95% CI)	IG (Advice + Application) Mean (95% CI)	CG (Advice) Mean (95% CI)
Vegetables (g/day)	294.4 ± 160.4	299.1 ± 171.1	12.0 (−4.0 to 8.1)	7.1 (−7.7 to 22.0)	−6.5 (−25.4 to 12.3)	5.6 (−13.2 to 24.3)
Fresh fruit (g/day)	356.3 ± 214.8	361.5 ± 235.2	14.9 (−12.6 to 42.4)	7.4 (−15.4 to 30.1)	14.0 (−14.5 to 42.5)	−1.3 (−27.9 to 25.3)
Whole-grains (g/day)	28.3 ± 58.5	26.2 ± 57.5	4.5 (−0.4 to 9.4)	−0.5 (−5.2 to 4.2)	0.7 (−5.3 to 6.8)	−1.0 (−6.3 to 4.3)
Pulses (g/day)	25.6 ± 15.2	25.1 ± 14.3	0.1 (−1.6 to 1.8)	−1.0 (−2.5 to 0.5)	2.1 (−0.16 to 4.3)	0.5 (−1.3 to 2.3)
Olive oil (g/day)	25.0 ± 16.0	24.2 ± 17.0	−1.3 (−3.0 to 0.5)	0.0 (−1.7 to 1.8)	0.8 (−1.3 to 2.9)	1.1 (−0.8 to 3.0)
Fish (g/day)	110.4 ± 59.3	116.7 ± 62.5	6.9 (−0.7 to 14.5)	4.9 (−3.6 to 13.3)	−3.4 (−10.5 to 3.8)	−1.5 (−8.8 to 5.8)
Nuts (g/day)	13.4 ± 18.9	14.3 ± 20.7	1.4 (−0.7 to 3.5)	1.9 (−0.3 to 4.0)	0.4 (−2.0 to 2.9)	1.0 (−1.2 to 3.3)
Dairy (g/day)	388.7 ± 209.9	379.6 ± 225.3	6.9 (−13.0 to 26.9)	−15.0 (−34.5 to 4.4)	−9.5 (−32.3 to 13.4)	1.7 (−20.6 to 23.9)
Industrial pastries (g/day)	32.1 ± 40.8	32.6 ± 46.0	−3.4 (−7.0 to 0.1)	−5.3 (−9.5 to −1.1) *	−4.0 (−8.3 to 0.3)	−1.9 (−6.8 to 2.9)
Red meat (g/day)	68.9 ± 47.0	66.6 ± 44.2	−4.3 (−9.0 to 0.5)	−6.3 (−10.9 to −1.7) **	−8.2 (−13.5 to −3.0) **	−5.7 (−10.4 to −1.0) *
White meat (g/day)	71.3 ± 49.1	65.8 ± 41.0	−2.0 (−8.2 to 4.2)	3.0 (−1.5 to 7.6)	−7.4 (−12.6 to −2.2) **	−1.1 (−5.4 to 3.2)
Processed meat (g/day)	36.5 ± 29.0	33.0 ± 24.7	−4.3 (−7.4 to −1.2) **	−4.6 (−6.8 to −2.4) **	−9.1 (−11.9 to −6.2) **	−3.0 (−5.7 to −0.4) *
Ready-made food (g/day)	14.6 ± 19.4	13.9 ± 19.2	−1.0 (−2.8 to 0.8)	−1.4 (−3.1 to 0.2)	−1.7 (−3.7 to 0.4)	−2.0 (−3.8 to −0.2) *

IG: intervention group; CG: control group; CI: confidence interval; * *p* < 0.05; ** *p* < 0.01.

**Table 5:** Effect of the intervention in terms of variation in food groups.

Food Group	Mean Difference (IG – CG) 3 Months		Mean Difference (IG – CG) 12 Months	
	Mean Difference (CI 95%)	*p*-Value	Mean Difference (CI 95%)	*p*-Value
Vegetables (g/day)	5.8 (−14.1 to 25.7)	0.569	−7.7 (−31.6 to 16.3)	0.529
Fresh fruit (g/day)	7.4 (−25.3 to 40.1)	0.655	16.2 (−19.1 to 51.5)	0.367
Whole-grains (g/day)	5.6 (−0.3 to 11.4)	0.063	2.0 (−4.1 to 8.2)	0.516
Pulses (g/day)	1.1 (−0.8 to 3.1)	0.257	1.7 (−0.8 to 4.3)	0.181
Olive oil (g/day)	−0.8 (−2.9 to 1.4)	0.482	0.4 (−2.0 to 2.7)	0.757
Fish (g/day)	−0.4 (−11.2 to 10.3)	0.940	−3.8 (−12.4 to 4.8)	0.392
Nuts (g/day)	−0.7 (−3.2 to 1.9)	0.613	−1.2 (−4.0 to 1.6)	0.407
Dairy (g/day)	21.6 (−3.4 to 46.6)	0.090	−7.7 (−36.0 to 20.7)	0.596
Industrial pastries (g/day)	0.8 (−3.2 to 4.9)	0.685	−2.9 (−7.9 to 2.0)	0.248
Red meat (g/day)	3.7 (−1.5 to 8.9)	0.165	−1.2 (−6.5 to 4.1)	0.663
White meat (g/day)	−1.2 (−7.8 to 5.5)	0.732	−3.4 (−8.5 to 1.6)	0.184
Processed meat (g/day)	2.4 (−0.7 to 5.5)	0.127	−4.3 (−7.1 to −1.5)	0.003
Ready-made food (g/day)	1.1 (−0.7 to 2.9)	0.218	0.4 (−1.3 to 2.2)	0.619

IG: intervention group; CG: control group; CI: confidence interval.

## Supplementary Materials

The following are available online at http://www.mdpi.com/2072-6643/10/10/1473/s1, Table S1: Baseline characteristics of the study population (completers vs non-completers).

## Availability of Data and Materials

The datasets used and/or analyzed during the current study are available from the corresponding author on reasonable request.

## Trial Registration

ClinicalTrials.gov Identifier: NCT02016014.

## Abbreviations

Abbreviation	Meaning
CG	counseling group
FFQ	food frequency questionnaire
IG	intervention group (counseling + application)

## References

[1] Forouhi N.G., Sattar N., Imamura F. (2017). Macronutrients and cardiovascular risk in a global context. Lancet Diabetes Endocrinol..

[2] Brown I.J., Elliott P., Robertson C.E., Chan Q., Daviglus M.L., Dyer A.R., Huang C.C., Rodriguez B.L., Sakata K., Ueshima H. (2009). Dietary starch intake of individuals and their blood pressure: The International Study of Macronutrients and Micronutrients and Blood Pressure. J. Hypertens..

[3] Lindseth G., Murray A. (2016). Dietary Macronutrients and Sleep. West. J. Nurs. Res..

[4] Doo M., Kim Y. (2016). Association between sleep duration and obesity is modified by dietary macronutrients intake in Korean. Obes. Res. Clin. Pract..

[5] Solfrizzi V., Custodero C., Lozupone M., Imbimbo B.P., Valiani V., Agosti P., Schilardi A., D’Introno A., La Montagna M., Calvani M. (2017). Relationships of Dietary Patterns, Foods, and Micro- and Macronutrients with Alzheimer’s Disease and Late-Life Cognitive Disorders: A systematic review. J. Alzheimers Dis..

[6] Estruch R., Ros E., Salas-Salvado J., Covas M.I., Corella D., Aros F., Gomez-Gracia E., Ruiz-Gutierrez V., Fiol M., Lapetra J. (2018). Primary Prevention of Cardiovascular Disease with a Mediterranean Diet Supplemented with Extra-Virgin Olive Oil or Nuts. N. Engl. J. Med..

[7] Turati F., Carioli G., Bravi F., Ferraroni M., Serraino D., Montella M., Giacosa A., Toffolutti F., Negri E., Levi F. (2018). Mediterranean Diet and Breast Cancer Risk. Nutrients.

[8] Bravi F., Spei M.E., Polesel J., Di Maso M., Montella M., Ferraroni M., Serraino D., Libra M., Negri E., La Vecchia C. (2018). Mediterranean Diet and Bladder Cancer Risk in Italy. Nutrients.

[9] Wright J.D., Wang C.Y. (2010). Trends in intake of energy and macronutrients in adults from 1999–2000 through 2007–2008. NCHS Data Brief.

[10] Vadiveloo M., Scott M., Quatromoni P., Jacques P., Parekh N. (2014). Trends in dietary fat and high-fat food intakes from 1991 to 2008 in the Framingham Heart Study participants. Br. J. Nutr..

[11] Su C., Zhao J., Wu Y., Wang H., Wang Z., Wang Y., Zhang B. (2017). Temporal Trends in Dietary Macronutrient Intakes among Adults in Rural China from 1991 to 2011: Findings from the CHNS. Nutrients.

[12] Eyles H., McLean R., Neal B., Doughty R.N., Jiang Y., Ni Mhurchu C. (2014). Using mobile technology to support lower-salt food choices for people with cardiovascular disease: Protocol for the SaltSwitch randomized controlled trial. BMC Public Health.

[13] Giacobbi P., Hingle M., Johnson T., Cunningham J.K., Armin J., Gordon J.S. (2016). See Me Smoke-Free: Protocol for a Research Study to Develop and Test the Feasibility of an mHealth App for Women to Address Smoking, Diet, and Physical Activity. JMIR Res. Protoc..

[14] Welch J.L., Astroth K.S., Perkins S.M., Johnson C.S., Connelly K., Siek K.A., Jones J., Scott L.L. (2013). Using a mobile application to self-monitor diet and fluid intake among adults receiving hemodialysis. Res. Nurs. Health.

[15] Stuckey M.I., Shapiro S., Gill D.P., Petrella R.J. (2013). A lifestyle intervention supported by mobile health technologies to improve the cardiometabolic risk profile of individuals at risk for cardiovascular disease and type 2 diabetes: Study rationale and protocol. BMC Public Health.

[16] Safran Naimark J., Madar Z., Shahar D.R. (2015). The impact of a Web-based app (eBalance) in promoting healthy lifestyles: Randomized controlled trial. J. Med. Internet Res..

[17] Coughlin S.S., Whitehead M., Sheats J.Q., Mastromonico J., Hardy D., Smith S.A. (2015). Smartphone Applications for Promoting Healthy Diet and Nutrition: A literature review. Jacobs J. Food Nutr..

[18] Allen J.K., Stephens J., Dennison Himmelfarb C.R., Stewart K.J., Hauck S. (2013). Randomized controlled pilot study testing use of smartphone technology for obesity treatment. J. Obes..

[19] Martin C.K., Miller A.C., Thomas D.M., Champagne C.M., Han H., Church T. (2015). Efficacy of SmartLoss^SM^, a smartphone-based weight loss intervention: Results from a randomized controlled trial. Obesity (Silver Spring).

[20] Duncan M., Vandelanotte C., Kolt G.S., Rosenkranz R.R., Caperchione C.M., George E.S., Ding H., Hooker C., Karunanithi M., Maeder A.J. (2014). Effectiveness of a web- and mobile phone-based intervention to promote physical activity and healthy eating in middle-aged males: Randomized controlled trial of the ManUp study. J. Med. Internet Res..

[21] Rabbi M., Pfammatter A., Zhang M., Spring B., Choudhury T. (2015). Automated personalized feedback for physical activity and dietary behavior change with mobile phones: A randomized controlled trial on adults. JMIR mHealth uHealth.

[22] Schoeppe S., Alley S., Van Lippevelde W., Bray N.A., Williams S.L., Duncan M.J., Vandelanotte C. (2016). Efficacy of interventions that use apps to improve diet, physical activity and sedentary behaviour: A systematic review. Int. J. Behav. Nutr. Phys. Act..

[23] Mummah S.A., Mathur M., King A.C., Gardner C.D., Sutton S. (2016). Mobile Technology for Vegetable Consumption: A Randomized Controlled Pilot Study in Overweight Adults. JMIR mHealth uHealth.

[24] Gilliland J., Sadler R., Clark A., O’Connor C., Milczarek M., Doherty S. (2015). Using a Smartphone Application to Promote Healthy Dietary Behaviours and Local Food Consumption. Biomed. Res. Int..

[25] Allman-Farinelli M., Partridge S.R., McGeechan K., Balestracci K., Hebden L., Wong A., Phongsavan P., Denney-Wilson E., Harris M.F., Bauman A. (2016). A Mobile Health Lifestyle Program for Prevention of Weight Gain in Young Adults (TXT2BFiT): Nine-month outcomes of a randomized controlled trial. JMIR mHealth uHealth.

[26] Elbert S.P., Dijkstra A., Oenema A. (2016). A Mobile Phone App Intervention Targeting Fruit and Vegetable Consumption: The efficacy of textual and auditory tailored health information tested in a randomized controlled trial. J. Med. Internet Res..

[27] Wharton C.M., Johnston C.S., Cunningham B.K., Sterner D. (2014). Dietary self-monitoring, but not dietary quality, improves with use of smartphone app technology in an 8-week weight loss trial. J. Nutr. Educ. Behav..

[28] Nollen N.L., Mayo M.S., Carlson S.E., Rapoff M.A., Goggin K.J., Ellerbeck E.F. (2014). Mobile technology for obesity prevention: A randomized pilot study in racial- and ethnic-minority girls. Am. J. Prev. Med..

[29] Hebden L., Cook A., van der Ploeg H.P., King L., Bauman A., Allman-Farinelli M. (2014). A mobile health intervention for weight management among young adults: A pilot randomised controlled trial. J. Hum. Nutr. Diet.

[30] Gilson N.D., Pavey T.G., Vandelanotte C., Duncan M.J., Gomersall S.R., Trost S.G., Brown W.J. (2016). Chronic disease risks and use of a smartphone application during a physical activity and dietary intervention in Australian truck drivers. Aust. N. Z. J. Public Health.

[31] Tonkin E., Brimblecombe J., Wycherley T.P. (2017). Characteristics of Smartphone Applications for Nutrition Improvement in Community Settings: A scoping review. Adv. Nutr..

[32] Recio-Rodriguez J.I., Martin-Cantera C., Gonzalez-Viejo N., Gomez-Arranz A., Arietaleanizbeascoa M.S., Schmolling-Guinovart Y., Maderuelo-Fernandez J.A., Perez-Arechaederra D., Rodriguez-Sanchez E., Gomez-Marcos M.A. (2014). Effectiveness of a smartphone application for improving healthy lifestyles, a randomized clinical trial (EVIDENT II): Study protocol. BMC Public Health.

[33] Garcia-Ortiz L., Recio-Rodriguez J.I., Martin-Cantera C., Cabrejas-Sanchez A., Gomez-Arranz A., Gonzalez-Viejo N., Iturregui-San Nicolas E., Patino-Alonso M.C., Gomez-Marcos M.A., the EVIDENT Group (2010). Physical exercise, fitness and dietary pattern and their relationship with circadian blood pressure pattern, augmentation index and endothelial dysfunction biological markers: EVIDENT study protocol. BMC Public Health.

[34] Consellería de Sanidade, Xunta de Galicia, España, Organización Panamericana de la salud (OPS-OMS) (2016). Epidat: Programa Para Análisis Epidemiológico de Datos.

[35] Prochaska J.O., Velicer W.F. (1997). The transtheoretical model of health behavior change. Am. J. Health Promot..

[36] Fernandez-Ballart J.D., Pinol J.L., Zazpe I., Corella D., Carrasco P., Toledo E., Perez-Bau M., Martinez-Gonzalez M.A., Salas-Salvado J., Martin-Moreno J.M. (2010). Relative validity of a semi-quantitative food-frequency questionnaire in an elderly Mediterranean population of Spain. Br. J. Nutr..

[37] Gupta S.K. (2011). Intention-to-treat concept: A review. Perspect Clin. Res..

[38] Vitale M., Masulli M., Rivellese A.A., Babini A.C., Boemi M., Bonora E., Buzzetti R., Ciano O., Cignarelli M., Cigolini M. (2016). Influence of dietary fat and carbohydrates proportions on plasma lipids, glucose control and low-grade inflammation in patients with type 2 diabetes-The TOSCA.IT Study. Eur. J. Nutr..

[39] Nettleton J.A., Rock C.L., Wang Y., Jenny N.S., Jacobs D.R. (2009). Associations between dietary macronutrient intake and plasma lipids demonstrate criterion performance of the Multi-Ethnic Study of Atherosclerosis (MESA) food-frequency questionnaire. Br. J. Nutr..

[40] Yamagishi K., Iso H., Yatsuya H., Tanabe N., Date C., Kikuchi S., Yamamoto A., Inaba Y., Tamakoshi A. (2010). Dietary intake of saturated fatty acids and mortality from cardiovascular disease in Japanese: The Japan Collaborative Cohort Study for Evaluation of Cancer Risk (JACC) Study. Am. J. Clin. Nutr..

[41] Gomez-Marcos M.A., Patino-Alonso M.C., Recio-Rodriguez J.I., Agudo-Conde C., Romaguera-Bosch M., Magdalena-Gonzalez O., Gomez-Arranz A., Mendizabal-Gallastegui N., Angel Fernandez-Diez J., Gomez-Sanchez L. (2018). Short- and long-term effectiveness of a smartphone application for improving measures of adiposity: A randomised clinical trial—EVIDENT II study. Eur. J. Cardiovasc. Nurs..

[42] Recio-Rodriguez J.I., Agudo-Conde C., Martin-Cantera C., Gonzalez-Viejo M.N., Fernandez-Alonso M.D., Arietaleanizbeaskoa M.S., Schmolling-Guinovart Y., Maderuelo-Fernandez J.A., Rodriguez-Sanchez E., Gomez-Marcos M.A. (2016). Short-Term Effectiveness of a Mobile Phone App for Increasing Physical Activity and Adherence to the Mediterranean Diet in Primary Care: A randomized controlled trial (EVIDENT II Study). J. Med. Internet Res..

[43] Wang Q., Egelandsdal B., Amdam G.V., Almli V.L., Oostindjer M. (2016). Diet and Physical Activity Apps: Perceived effectiveness by app users. JMIR mHealth uHealth.

