# Supplementary material for: The Effectiveness of a Smartphone Application on Modifying the Intakes of Macro and Micronutrients in Primary Care: A Randomized Controlled Trial. The EVIDENT II Study
Source: Nutrients. 2018 Oct 10;10(10):1473. doi: 10.3390/nu10101473 (PMC6212958; doi:10.3390/nu10101473)
Supplement: Supplementary file 1 [file nutrients-10-01473-s001.pdf]

Table S1: Baseline characteristics of the study population (completers vs non-completers)

		Completers (715; 85.8%)		Non completers (118; 14.2%)		
Variable		Mean/N	SD/ (%)	Mean/N	SD/ (%)	p value
Age (years)		52.3	11.7	49.3	13.7	0.012
Females (n, %)		439	61.4	78	66.1	0.329
Work situation (n, %)	Works outside of home	362	50.6	56	47.5	0.021
	Homemaker	109	15.2	16	13.5	
	Retired	138	19.3	18	15.3	
	Student	10	1.5	8	6.8	
	Unemployed	96	13.4	20	16.9	
Educational level (n, %)	University studies	211	29.5	38	32.2	0.170
	Middle or High school	367	51.3	49	41.5	
	Elementary school	137	19.2	31	26.3	
Smoking (n, %)	Non smoker	308	43.1	48	40.7	0.213
	Smoker	166	23.2	36	30.5	
	Former smoker	241	33.7	34	28.8	
BMI mean (Kg/m2)		27.8	4.7	28.2	5.7	0.416
BMI Categories (n, %)	BMI<25	213	29.8	35	29.7	0.358
	BMI 25-30	302	42.2	43	36.4	
	BMI > 30	200	28	40	33.9	
Systolic blood pressure (mmHg)		123.9	16.2	124.0	16.0	0.928
Diastolic blood pressure (mmHg)		76.2	9.8	76.2	10.4	0.940
Total Cholesterol (mg/dl)		203.9	35.6	205.0	39.5	0.762
Glycated Haemoglobin (%)		5.5	0.5	5.6	0.9	0.100
Physical activity						
Counts minute/week		69.9	68.4	59.7	74.9	0.162
METS/ minute /week		1871.0	891.3	1652.5	948.2	0.021

IG: Intervention group; CG: Control group; BMI: Body mass index; METS: Metabolic Equivalent of Task
